# Determination of anxiety, mood disorders and disability in cluster and migraine headache

**DOI:** 10.1186/1129-2377-14-S1-P103

**Published:** 2013-02-21

**Authors:** C Gonzalez, S Benitez, T Gomez, M Bernal, MD Jimenez

**Affiliations:** 1 Virgen del Rocio Universitary Hospital, Spain

## Introduction

Headache is the most frequent pathology in neurology. Migraine is very frequent in the population and it is a disease of young people, with repercussion in their social life and in their job and with important expenses. In cluster headache the pain is very severe and disabling. The patients have difficulties in having a normal life and are often absent in their jobs – some of them even lose their employment – and very frequently use symptomatic and very expensive treatments. All of the above mentioned causes severe anxiety, mood disorders and disabilities to these patients.

## Method and objectives

In order to quantify those effects and to determine quantitative measures of depression, anxiety and disability we applied the “Hamilton Depression rating Scale” test and the “Hamilton Anxiety Rating Scale”, a generic instrument for determining quality of life “SF36-2” and a headache specific instrument of disability “MIDAS test”. We study 54 patients with cluster (17 chronic cluster and 37 episodic cluster) and 80 with migraine (40 chronic migraine and 40 episodic migraine). Result: Anxiety and depression are more important in chronic migraine and cluster than in episodic forms. MIDAS test results are more affected in chronic forms due to the higher frequency of headache episodes. In the SF36, we can see that these patients have disability in multiple fields, more important in chronic forms. We compare every field in two different kind of headache.

## Conclusion

Cluster and migraine are diseases very disabling and with repercussion in the patients life, specially in chronic forms. They lose jobs and money, with very important consequencies for them and their families. It is important to recognize these symptoms to offer better therapies and multidisciplinary management to our patients.

## References

[B1] Abbass A Lovas D Purdy A Direct diagnosis and management of emotional factors in chronic headache patients Cephalalgia 2008 14 12 1305 14 10.1111/j.1468-2982.2008.01680.x 18771494

[B2] Jürgens TP Gaul C Lindwurm A Dresler T Paelecke-Habermann Y Schmidt-Wilcke T Lürding R Henkel K Leinisch E Impairment in episodic and chronic cluster headache Cephalalgia 2011 14 6 671 82 10.1177/0333102410391489 21123629

